# Supplementary material for: OpenMM 7: Rapid development of high performance algorithms for molecular dynamics
Source: PLoS Comput Biol. 2017 Jul 26;13(7):e1005659. doi: 10.1371/journal.pcbi.1005659 (PMC5549999; doi:10.1371/journal.pcbi.1005659)
Supplement: S1 Source Code — (ZIP) [file pcbi.1005659.s002.zip › OpenMM-7.0.1-Source/docs/api-python/generated/simtk.openmm.app.checkpointreporter.CheckpointReporter.html]

CheckpointReporter — OpenMM 7.0.1.dev-5e86c4f documentation


# CheckpointReporter¶

*class* `simtk.openmm.app.checkpointreporter.``CheckpointReporter`(*file*, *reportInterval*)¶
:   CheckpointReporter saves periodic checkpoints of a simulation.
    The checkpoints will overwrite one another – only the last checkpoint
    will be saved in the file.

    To use it, create a CheckpointReporter, then add it to the Simulation’s
    list of reporters. To load a checkpoint file and continue a simulation,
    use the following recipe:

    ```
    >>> with open('checkput.chk', 'rb') as f:
    >>>     simulation.context.loadCheckpoint(f.read())
    ```

    Notes:
    A checkpoint contains not only publicly visible data such as the particle
    positions and velocities, but also internal data such as the states of
    random number generators. Ideally, loading a checkpoint should restore the
    Context to an identical state to when it was written, such that continuing
    the simulation will produce an identical trajectory. This is not strictly
    guaranteed to be true, however, and should not be relied on. For most
    purposes, however, the internal state should be close enough to be
    reasonably considered equivalent.

    A checkpoint contains data that is highly specific to the Context from
    which it was created. It depends on the details of the System, the
    Platform being used, and the hardware and software of the computer it was
    created on. If you try to load it on a computer with different hardware,
    or for a System that is different in any way, loading is likely to fail.
    Checkpoints created with different versions of OpenMM are also often
    incompatible. If a checkpoint cannot be loaded, that is signaled by
    throwing an exception.

    `__init__`(*file*, *reportInterval*)¶
    :   Create a CheckpointReporter.

        |  |  |
        | --- | --- |
        | Parameters: | - **file** (*string or open file object*) – The file to write to. Any current contents will be overwritten. - **reportInterval** (*int*) – The interval (in time steps) at which to write checkpoints. |

    Methods

    |  |  |
    | --- | --- |
    | `__init__`(file, reportInterval) | Create a CheckpointReporter. |
    | `describeNextReport`(simulation) | Get information about the next report this object will generate. |
    | `report`(simulation, state) | Generate a report. |

    `describeNextReport`(*simulation*)¶
    :   Get information about the next report this object will generate.

        |  |  |
        | --- | --- |
        | Parameters: | **simulation** (*Simulation*) – The Simulation to generate a report for |
        | Returns: | A five element tuple. The first element is the number of steps until the next report. The remaining elements specify whether that report will require positions, velocities, forces, and energies respectively. |
        | Return type: | tuple |

    `__delattr__`¶
    :   x.\_\_delattr\_\_(‘name’) <==> del x.name

    `__format__`()¶
    :   default object formatter

    `__getattribute__`¶
    :   x.\_\_getattribute\_\_(‘name’) <==> x.name

    `__hash__`¶

    `__reduce__`()¶
    :   helper for pickle

    `__reduce_ex__`()¶
    :   helper for pickle

    `__repr__`¶

    `__setattr__`¶
    :   x.\_\_setattr\_\_(‘name’, value) <==> x.name = value

    `__sizeof__`() → int¶
    :   size of object in memory, in bytes

    `__str__`¶

    `report`(*simulation*, *state*)¶
    :   Generate a report.

        |  |  |
        | --- | --- |
        | Parameters: | - **simulation** (*Simulation*) – The Simulation to generate a report for - **state** (*State*) – The current state of the simulation |

High performance molecular simulation on GPUs


### Navigation

- Application Layer
  - Loaders and Setup
  - Representation and Manipulation
  - Simulation
  - Reporting Output
  - Extras
- Library Layer

©2015, Stanford University and the Authors.
|
Powered by Sphinx 1.3.1
& Alabaster 0.7.7
|
Page source
